# Aerobic exercise training and burnout: a pilot study with male participants suffering from burnout

**DOI:** 10.1186/1756-0500-6-78

**Published:** 2013-03-04

**Authors:** Markus Gerber, Serge Brand, Catherine Elliot, Edith Holsboer-Trachsler, Uwe Pühse, Johannes Beck

**Affiliations:** 1 Institute of Exercise and Health Sciences, University of Basel, Basel, Switzerland; 2 Psychiatric Hospital of the University of Basel, Center for Affective, Stress and Sleep Disorders, Basel, Switzerland

**Keywords:** Aerobic exercise, Burnout, Depressive symptoms, Maslach Burnout Inventory, Mood, Stress

## Abstract

**Background:**

Occupational burnout is associated with severe negative health effects. While stress management programs proved to have a positive influence on the well-being of patients suffering from burnout, it remains unclear whether aerobic exercise alleviates burnout severity and other parameters related to occupational burnout. Therefore, the main purpose of this study was to pilot-test the potential outcomes of a 12-week exercise training to generate hypotheses for future larger scale studies.

**Methods:**

The sample consisted of 12 male participants scoring high on the MBI emotional exhaustion and depersonalization subscales. The training program took place in a private fitness center with a 17.5 kcal/kg minimum requirement of weekly energy expenditure.

**Results:**

The key findings are that increased exercise reduced overall perceived stress as well as symptoms of burnout and depression. The magnitude of the effects was large, revealing changes of substantial practical relevance. Additionally, profiles of mood states improved considerably after single exercise sessions with a marked shift towards an iceberg profile.

**Conclusion:**

Among burnout patients, the findings provide preliminary evidence that exercise has the potential to reduce stress and prevent the development of a deeper depression. This has important health implications given that burnout is considered an antecedent of depressive disorders.

**Trial registration:**

ClinicalTrials.gov Identifier: ISRNCT01575743

## Background

The term burnout is used to describe a gradual depletion of energy combined with a loss of motivation and commitment after prolonged exposure to high occupational stress [1]. Examples of occupational stress include high workload, role conflicts, lack of participation or social support, injustice, uncertainty, under-reward, ambiguity, job insecurity, job complexity, as well as structural constraints [2-5].

Several validated instruments exist to assess burnout in working populations. Nevertheless, the most frequently used questionnaire is the MBI and its adaptations [1,6]. Maslach and colleagues [7] operationalized burnout as a three-dimensional concept encompassing emotional exhaustion, depersonalization/cynicism, and reduced personal accomplishment. Emotional exhaustion describes feelings of depleted emotional resources, overstrain, tiredness, or fatigue and constitutes the individual energy component of the syndrome. Depersonalization/cynicism represents the interpersonal dimension of burnout which is characterized by negative, cynical, or excessively indifferent, distant and detached responses to other people at work and is associated with lack of work-related enthusiasm. Finally, reduced personal accomplishment contains a strong self-evaluative component and describes a drop in perceived competence, professional self-efficacy and productivity [7].

For several reasons, burnout presents an important public health problem and a cause of concern for policy makers [5]. Schaufeli and Enzmann [4] observed a considerable rise of stress-related compensation claims in various advanced economies. They further estimated that between 4% and 8% of the working population experience severe burnout. Ahola et al. [8] estimated the prevalence of mild and severe burnout in an epidemiologic study at 25% and 2.4%, respectively. Furthermore, research has revealed a transfer of burnout symptoms from one employee to another [9]. Consequently, burnout may negatively impact the work atmosphere within a company or institution. Moreover, burnout has a high temporal stability. Across various occupational groups and cultural contexts, test-retest correlations varied between .50 and .60 even if participants were examined over several years [10,11].

With respect to the consequences of occupational burnout, researchers found associations between high burnout and increased labour turnover and absenteeism rates [12], reduced organizational commitment [13] and lower job performance [14]. Additionally, previous investigations have shown that burnout negatively affects both aspects of mental and physical health. For instance, researchers have observed significant associations between burnout and increased depressive symptoms [15], as well as poor sleep and chronic fatigue syndrome [16-19]. Moreover, research has shown that burnout is linked with poorer self-rated health [20], and a variety of somatic complaints such as headaches and gastro-intestinal problems [21]. Finally, both cross-sectional case–control studies and prospective investigations have shown that burnout is related to increased risk of cardiovascular disease related events with a relative risk comparable to other risk factors such as body mass index, smoking, blood pressure, and lipid levels [2].

In summary, the findings presented above indicate that the number of people unable to work due to mental difficulties has increased during recent decades and that burnout may be a vital component in this development. Consequently, knowledge about burnout prevention and efficient treatment are pivotal issues in the public health and economic perspectives. Previous intervention programs have focused on improving stress and emotion management techniques [4]. There are several legitimate reasons for stress intervention programs. First, chronic stress is a key factor in the causality of burnout. Second, negative coping strategies contribute to the maintenance and aggravation of burnout symptoms. Third, both stress and burnout have been linked with a dysregulation of the hypothalamic-pituitary-adrenocortical (HPA) axis [22].

The main purpose of the present study was to pilot-test the potential of aerobic exercise training as a simple and inexpensive alternative stress treatment. So far, some studies have investigated the relationship between self-reported exercise and occupational burnout in general population samples [23-28], but no studies have been conducted that relate to whether a structured aerobic exercise training is able to reduce occupational burnout and associated symptoms of psychopathology among individuals with high initial burnout scores. Nevertheless, this is a pertinent issue because previous research has established links between high exercise and decreased levels of perceived stress [29-31]. Additionally, frequent exercise protects against impaired health if individuals are exposed to high stress demands [for review: [32]. Leisure time physical activity has been shown to buffer work-related stress [33,34], while randomized controlled trials have revealed that exercise interventions can reduce overall perceived stress [35]. Additionally, evidence exists that exercise has positive effects in the treatment of people suffering from depressive disorders [36-39]. Studies have also revealed that aerobic exercise training leads to enhanced mood [40]. Specifically, researchers observed that aerobic exercise results in mood states resembling an ‘iceberg profile’, which consists of elevated scores on the vigour subscale combined with low scores on depression, anger, confusion, fatigue and tension [41].

### Purpose

The main purpose of the present pilot study was to explore whether a three-month aerobic exercise training program results in a) reduced levels of burnout, b) decreased depressive symptoms, and c) reduced stress perceptions. Furthermore, the present study aims to examine whether single exercise training sessions can improve mood, that is whether mood states will change in the direction of an ‘iceberg’ profile characterized by high vigour and low negative emotion states.

## Methods

### Participants and procedures

The sample consisted of twelve men (M = 45.8 years, SD = 6.8; range 36–65 years) suffering from burnout syndrome based on Maslach’s definition of occupational burnout [7]. All participants continued working during study participation. Participants were approached through different channels. Some participants were contacted via email because they completed a burnout questionnaire on a public website (http://www.swissburnout.ch), others received flyers from their general practitioners and psychiatrists, and some responded to public advertisements in hospitals, firms, offices and through publication in several discussion forums (e.g. http://forum.swissburnout.ch). All participants were screened via telephone interview prior to study enrolment by an experienced rater to ensure that all participants had high levels of work-related burnout. The following inclusion criteria were applied: a) high scores on the MBI subscales of ‘emotional exhaustion’ (≥ 27) or ‘depersonalization’ (≥ 10), male gender, aged between 30 to 65 years, non-smoking, good physical health, and not involved in regular exercise during the last two years. Subjects were excluded if they suffered from neurological illnesses, metabolic illnesses, liver or renal dysfunction, acute or chronic infectious diseases, neoplasias, or if they received pharmacotherapy or psychotherapy during the study.

### Study design

This one-group pilot study was designed as a pre-experimental, pretest-posttest investigation. All participants underwent a baseline examination one week before they started the 12-week exercise-training program. The examination used standard questionnaires to assess burnout severity, depressive symptoms and perceived stress. Baseline testing occurred under supervision of an experienced investigator who assisted the participants in the case of ambiguity. After baseline examination, all participants engaged in a 12-week exercise training at a private fitness centre under supervision of exercise coaches from the Institute of Exercise and Health Sciences at the University of Basel. Three days after successful completion of the exercise program, participants answered the same questionnaires under identical conditions. Additionally, six randomly selected participants answered a mood questionnaire on two occasions (week 5 and 9) immediately before and after the training session.

Prior to baseline testing, detailed information was given to all participants about the purpose of the study and the exercise program. Informed written consent was required and participants were aware that participation is voluntary and that they can discontinue participation at any time. The local ethics committee of Basel (EKBB) approved the study protocol and the investigation was conducted in accordance with the principles of the Declaration of Helsinki.

### Exercise training

In accordance with the study by Dunn et al. [37], the aerobic exercise program was based on the exercise prescription guidelines of the American College of Sports Medicine [42]. The required level of weekly energy expenditure was 17.5 kcal/kg. Energy expenditure was assessed using the values of burned calories provided by the training devices based on age, weight and training performance. Only the total weekly amount was used as a criterion. Accordingly, participants could choose their exercise frequency (between 2–3 trainings per week) and intensity. To allow for varied training and to avoid symptoms of physical overload, participants had a choice of different ergometers such as a cross trainer (Nautilus E916), a running ergometer (TechnoGym JogEcite), a stepping ergometer (TreadClimer®TC919), a bicycle ergometer (Life Fitness 95ci) and a rowing ergometer (Concept 2 Model E Indoor Rower P). Typically, participants exercised for about one hour, either two or three days per week. Participants were instructed to exercise within 60-75% of their maximum heart rate. The maximum heart rate was estimated according to the ACSM guidelines [42] using the formula: maximum heart rate = 220 – age (years) for untrained individuals. Heart rate was monitored during all training sessions with a chest belt heart rate monitor (Polar®) to ensure training below the anaerobic threshold. Training intensity was adjusted at baseline and after 6 weeks of training using the YMCA-test [43]. Participants were instructed to engage only in physical activity during the exercise intervention that they performed on a regular basis prior to the beginning of the program (e.g. cycling to work).

### Instruments

Burnout symptoms: Work-related burnout was measured with a validated German version of the Maslach Burnout Inventory [1]. The MBI consisted of 22 items assessing the three sub-dimensions of emotional exhaustion (9 items; Cronbach’s alpha = .84 [baseline] and .88 [follow-up]), depersonalization (5 items: Cronbach’s alpha = .89 [baseline] and .76 [follow-up]) and personal accomplishment (8 items: Cronbach’s alpha = .89 [baseline] and .88 [follow-up]). Responses were given on a 7-point frequency rating scale, ranging between 0 (never) to 6 (every day). Burnout was represented by high scores on emotional exhaustion and depersonalization, and low scores on personal accomplishment. The three-factor structure of all versions of the MBI have been confirmed in several empirical studies across samples from different countries [44]. These investigations revealed that the three dimensions are conceptually distinct, but empirically related. Researchers have made recommendations against using an aggregate of the three MBI components [7].

Depressive symptoms: The Beck Depression Inventory [BDI: [45] was used to assess the severity of depressive symptoms. The BDI consists of 21 items including a range of affective, behavioral, cognitive, and somatic symptoms that are indicative of unipolar depression (e.g. ‘I am so unhappy/sad that I can’t stand it’). Participants were asked to select from four responses that reflect increasing levels of depressive symptomatology. Possible scores ranged from 0 to 63 with higher scores indicating more depressive symptoms. Adequate validity and reliability of the BDI have been shown previously [46]. The Cronbach’s alpha in the present study was α = .85 (baseline) and α = .87 (follow-up).

Perceived stress: Overall perceived stress was assessed with the Perceived Stress Scale [PSS: [47], which is among the most widely used instruments for measuring perceived stress. The PSS consists of 10 items and draws on cognitive-transactional stress theory. Thus, the PSS measures the degree to which respondents find their lives unpredictable, uncontrollable, and overloading (e.g., ‘How often have you felt that you could not control the important things in your life?’). Answers were given on a 5-point Likert-scale anchored at 1 (*never*) to 5 (*very often*). Four items were reverse scored. Mean scores were calculated. Adequate validity and reliability of the PSS have been previously established [48]. The Cronbach’s alpha in the present study was α = .71 (baseline) and α = .86 (follow-up).

Mood: Current mood states were assessed with a multidimensional instrument, the Befindlichkeitsskala [BFS: [49]. Prior research has indicated adequate validity and reliability of the BFS [49]. The BFS contains two bipolar core dimensions (high/low tension, negative/positive evaluation). The instrument consists of an adjective list for self-rating current psychophysiological mood states. The 40 items are answered on a Likert-scale ranging from 1 (*not at all*) to 5 (*very much*) and represent eight subscales with five items each. The dimensions are labelled as: anger (Cronbach’s alpha = .73 - .87), excitation (Cronbach’s alpha = .79 - .90), activation (Cronbach’s alpha = .74 - .95), elation (Cronbach’s alpha = .93 - .95), calmness (Cronbach’s alpha = .90 - .91), contemplativeness (Cronbach’s alpha = .62 - .64), fatigue (Cronbach’s alpha = .62 - .80), depression (Cronbach’s alpha = .93 - .94).

### Statistical analysis

Changes in mean scores within the sample from pre to post exercise training were tested with paired-sample t-tests. All statistical calculations were performed with SPSS 18.0 for Apple Mac®. Results with an alpha level below .05 were considered statistically significant. Given the small sample size involved in this pilot study, we also calculated effect sizes (*d*) to interpret the meaningfulness of the data. Following Cohen’s [50] recommendations, 0.20 ≥ *d* ≥ 0.49 was considered to be small (e.g. negligible practical importance), 0.50 ≥ *d* ≥ 0.79 was medium (e.g. moderate practical importance), and *d* ≥ 0.80 was large (e.g. crucial practical importance).

## Results

**Figure 1:**
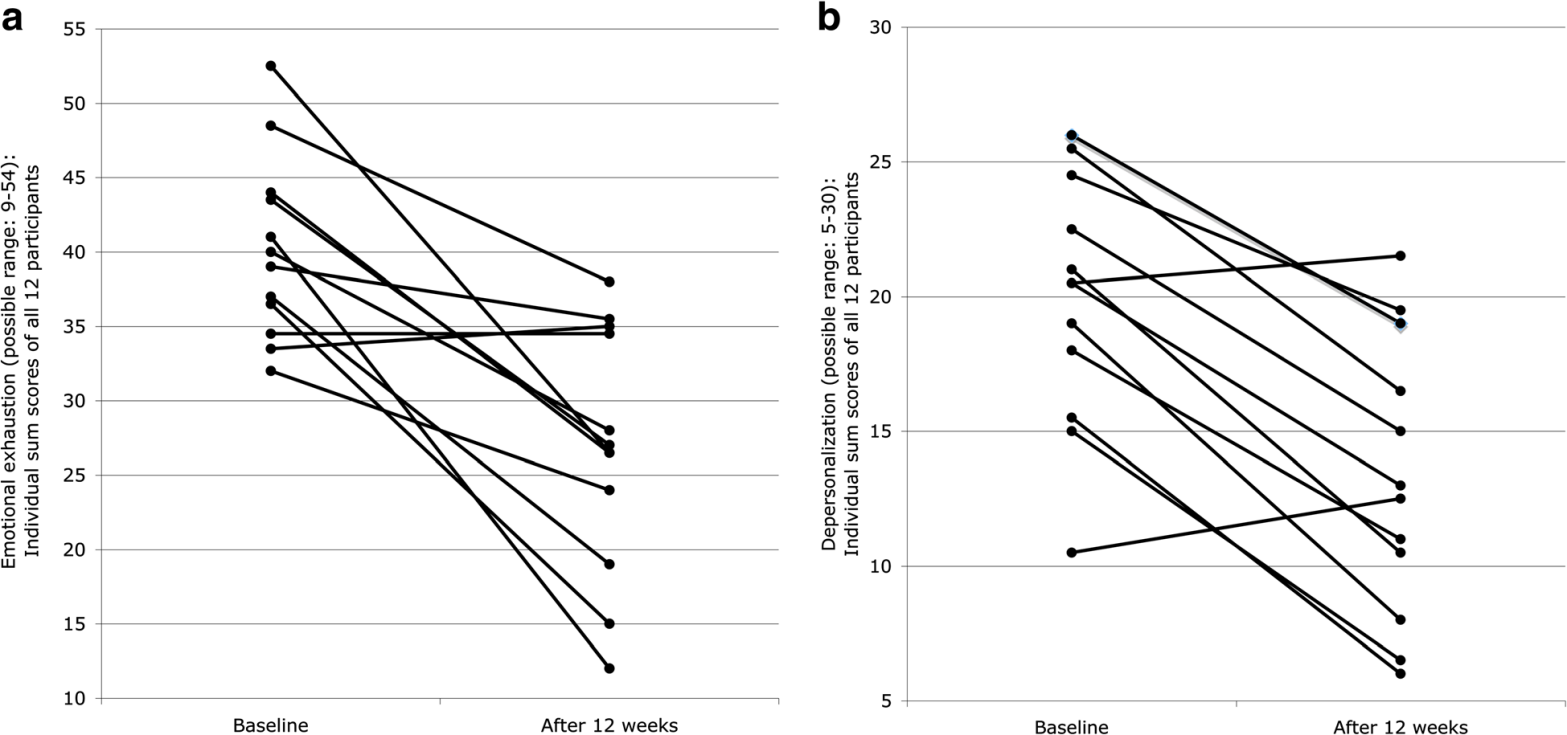
**Participants’ Changes in Burnout Symptomatology After Twelve Weeks Aerobic Exercise Training Regarding Emotional Exhaustion (Figure **1**a) and Depersonalization (Figure **1**b).**

Participants significantly reduced their burnout symptoms after the 12-week aerobic exercise program. As presented in Table 1, participants reduced their feelings of emotional exhaustion, while concurrently experiencing less depersonalization. No significant changes were found with regard to personal accomplishment. Regarding the magnitude of the changes, large effect sizes were found for emotional exhaustion (*d* = 1.84) and depersonalization (*d* = 1.35). In turn, the scores for personal accomplishment pointed towards a change of negligible practical importance (*d* = 0.31). Figure 1a and 1b show that ten of twelve participants reduced their emotional exhaustion and depersonalization scores, most of them to a considerable degree.

**Table 1:** Contrast of time 1 (Baseline) with time 2 (After 12 weeks exercise training) regarding psychopathological symptoms and stress

	**Time 1**		**Time 2**				**Cohen’s *d***
	***M***	***SD***	***M***	***SD***	***t*(11)**	***p***	
Emotional exhaustion	40.18	6.15	26.75	8.29	4.75	< .001	1.84
Depersonalization	19.86	4.62	13.25	5.16	5.54	< .001	1.35
Personal accomplishment	30.47	7.47	34.50	5.49	−1.64	*ns*	0.31
Depressive symptoms	17.58	8.28	7.42	4.91	4.72	< .001	1.54
Perceived stress	26.50	3.78	17.92	5.95	4.50	< .001	1.76

**Figure 2:**
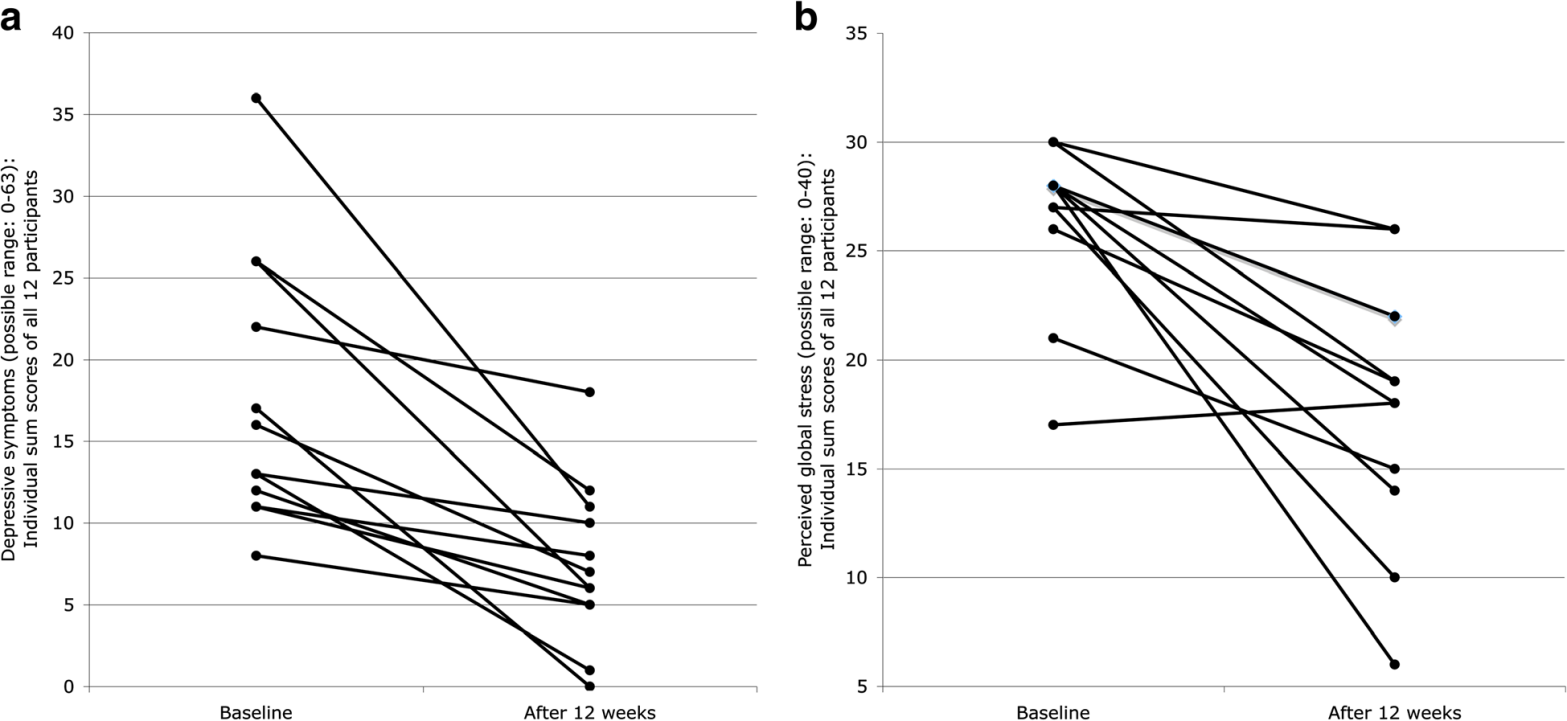
**Participants’ Changes in Depressive Symptoms (Figure **2**a) and Perceived Global Stress (Figure **2**b) After Twelve Weeks Aerobic Exercise Training.**

A significant reduction was also observed for depressive symptoms, with an effect size indicating a large change from baseline to follow-up (*d* = 1.54; Table 1). Figure 2a shows that all participants reduced their scores on the BDI over time.

Participants also significantly reduced their levels of perceived stress, with a large effect size for the intervention program (*d* = 1.76; Table 1). As Figure 2b shows, eleven of the twelve participants decreased their stress scores. Only one participant with a relatively low initial score maintained his level of previous stress.

In summary, our exploratory data suggest that a 12-week exercise program has the potential to reduce symptoms of occupational burnout, symptoms of depression and levels of perceived stress among male participants with high initial burnout scores.

**Figure 3:**
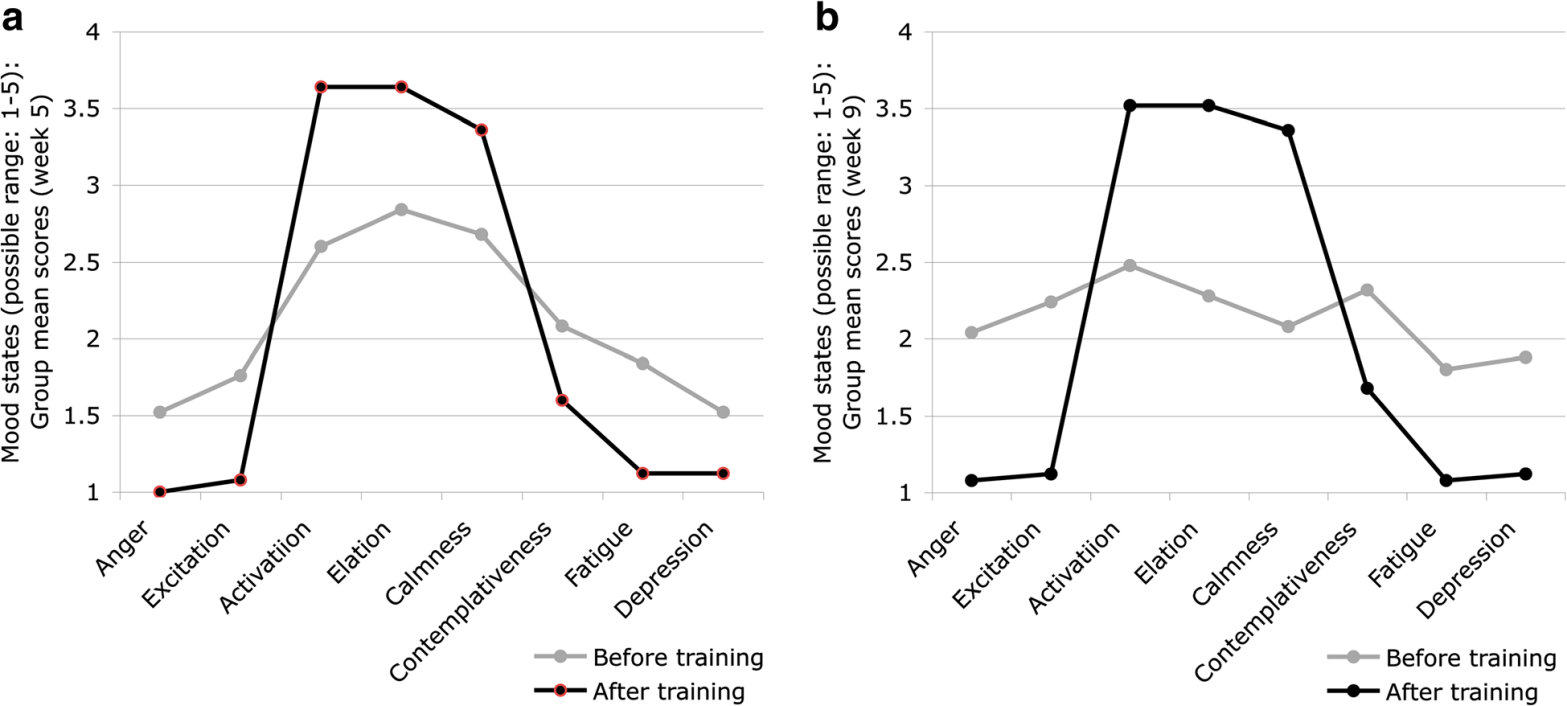
**Changes in Mood States From Before to After a Single Exercise Session at Week 5 (Figure **3**a) and Week 9 (Figure **3**b).**

The results regarding mood changes from before to after a single training session are summarized in Table 2 and 3. As illustrated in Figures 3a and 3b, participants’ profiles of mood states clearly shifted towards an ‘iceberg profile’. Although changes in most mood sub-dimensions were below the level of statistical significance, a closer inspection of effect sizes (between *d* = |-0.60| and |-2.00|) revealed that the changes are indeed meaningful.

**Table 2:** Contrast of time 1 (Before training session) with time 2 (After training session) regarding mood states at week 5

	**Time 1**		**Time 2**				**Cohen’s *d***
	***M***	***SD***	***M***	***SD***	***t*(11)**	***p***	
Anger	1.52	1.05	1.00	0.00	1.10	*ns*	0.99
Excitation	1.76	0.96	1.08	0.11	1.51	*ns*	1.27
Activatiion	2.6	1.03	3.64	0.89	−1.67	*ns*	−1.80
Elation	2.84	1.16	3.64	1.06	−1.09	*ns*	−0.72
Calmness	2.68	1.25	3.36	1.03	−0.85	*ns*	−0.60
Contemplativeness	2.08	0.72	1.6	0.55	1.11	*ns*	0.76
Fatigue	1.84	0.84	1.12	0.27	1.88	*ns*	1.30
Depression	1.52	0.64	1.12	0.18	1.45	*ns*	0.98

**Table 3:** Contrast of time 1 (Before training session) with time 2 (After training session) regarding mood states at week 9

	**Time 1**		**Time 2**				**Cohen’s *d***
	***M***	***SD***	***M***	***SD***	***t*(11)**	***p***	
Anger	2.04	1.48	1.08	0.11	1.40	*ns*	1.21
Excitation	2.24	1.05	1.12	0.11	2.22	*ns*	1.93
Activatiion	2.48	0.44	3.52	0.58	−2.89	< .05	−1.42
Elation	2.28	0.58	3.52	0.66	−2.74	*ns*	−2.00
Calmness	2.08	0.80	3.36	0.99	−2.43	*ns*	−1.43
Contemplativeness	2.32	0.42	1.68	0.23	2.67	*ns*	1.97
Fatigue	1.80	0.91	1.08	0.18	1.67	*ns*	1.32
Depression	1.88	1.34	1.12	0.18	1.22	*ns*	1.00

## Discussion

The key findings of the present pilot study were that a 12-week aerobic exercise program successfully reduced symptoms of burnout and depression in a sample of male participants scoring high on the MBI emotional exhaustion and depersonalization scales. Twelve weeks of exercise training also led to a significant reduction in perceived stress. All effect sizes were large, indicating changes of substantive significance. In addition, mood states improved considerably from before to after a single exercise session.

In a pilot-study with only 12 participants, our ambition was not to test hypotheses. Rather, this investigation was designed to generate empirical evidence, on which hypotheses can be based in future, large-scale studies. Furthermore, the effect sizes observed in this pilot study can be used to estimate the optimal sample size in forthcoming randomized controlled trials.

Melamed et al. [2] argued that the therapy of burnout is a challenging endeavour, as the success of many interventions may be limited by the fact that later stages of burnout entail physiological changes that are not easily reversed [16,51]. Previous research has shown that aerobic exercise is negatively associated with symptoms of burnout [23-28], is suited to reduce perceived stress [29-31], and improves biological markers that may mediate between burnout and cardiovascular disease (i.e. sleep and immunology) [2]. Drawing on recent findings in depressive disorder research [36-39], positive effects of aerobic exercise training with burnout patients were hypothesized. Nonetheless, burnout and depression are separate, yet overlapping concepts. First, perceived fatigue and low levels of energy are a core component in all burnout measures and instruments to assess major depressive disorder [52]. Second, there are similarities between depersonalization, social withdrawal and learned helplessness, which are also involved in depression [46]. Conceptually, the two constructs are different in that depression is a global (context-free) affective state, whereas burnout is perceived in the work environment [9]. Accordingly, Schaufeli and Enzmann [4] showed that emotional exhaustion and depression shared only 26% of variance, while the degree of overlap was smaller for depersonalization (13%) and personal accomplishment (9%).

Since no prior research existed regarding the impact of aerobic exercise training among participants with high burnout scores, a pilot study seemed warranted. Despite the small sample size, our study provides promising and encouraging results in the sense that significant and clinically relevant changes were observed in almost all variables and in almost all participants. There were several reasons for these findings. First, participants might have perceived aerobic exercise as a burnout remedy. In particular, exercise is considered acceptable as a treatment against depression among adults, with a perceived effectiveness comparable to antidepressants and cognitive-behaviour therapy [53]. Second, aerobic exercise training might have contributed to improved sleep and recovery, which could have resulted in a decrease in emotional exhaustion [54]. Third, the close contact with the exercise trainers and the promotion of personal and social resources might have contributed to the reduction in depersonalization due to increased social support. Shirom et al. [5] argued that burnout is linked to a depletion of work-related resources, and that fostering resources that build up self-efficacy, hardiness and social support may provide positive results. We assume that aerobic exercise, as implemented in the present study, was well suited to improve these key resources. No improvements were found in the personal accomplishment subscale of the MBI. Exercise training did not directly influence the working-conditions of the participants. Therefore, it is possible for feelings of satisfaction to remain low in a climate of high job-pressures, low autonomy and lack of recognition.

It is also possible that re-occurring experiences of positive mood states may help to disrupt negative thinking cycles and thus prohibit the progression of cognitive downward spirals [55]. As noted by Terry [41], the “link between physical activity and mood is perhaps one of the most intuitively appealing relationships in the whole area of sport and exercise psychology. (…) However, intuitive appeal and empirical support is not the same thing” (p. 1). This study provides empirical support that moderate intensity exercise generally improves mood states of participants with high burnout scores.

One strength of the present study was the relatively homogeneous sample that diminished the influence of confounding factors. Additionally, participants were selected on the basis of high MBI scores and had not regularly exercised for at least twelve months prior to the exercise intervention. Another strength was that exercise training took place in a natural setting (private fitness centre with choice of ergometer) under controlled circumstances (supervised by experienced exercise trainers). This ensured that all participants fulfilled the a priori exercise requirements (17.5 kcal/kg body weight per week, between 60-75% of maximal heart rate). Additional strengths were that there was no participant mortality, that only validated measures were used to assess psychological variables, and that *p*-values, effect sizes, group means and individual scores were used to interpret the data.

Despite these strengths, some important limitations hindered the generalization of the findings. First, the study was designed as a one-group, pre-post-test investigation with no control group, placebo control group (e.g. performing table games) or waiting-list control group. With regard to internal validity, this design was relatively weak, only allowing observation of changes occurring within the intervention group. For instance, values might have changed naturally over time, or from events that took place during the treatment period. Since the intervention group started with relatively high levels of burnout, a regression to the mean seems plausible. Another important limitation was the small sample size. However, several depressive disorder studies [36-39] showed that it was apropos to assume a strong influence (*d* ≥ 0.80). Thus, with an alpha set at .05 and a power of .80 [47], twelve participants constituted a sufficiently large sample and allowed dependent *t*-test calculations. It remains uncertain whether exercise in a group setting would have resulted in different findings than a one-on-one supervision model. Also, the findings have limited generalizability since the sample included only non-smoking males with low levels of mental and physical co-morbidities who were not receiving pharmacotherapy or psychotherapy at the time of the study. Regarding the exercise program, limitations were that only prediction equations, although validated, were used to establish energy expenditure and maximum heart rate and that physical activity outside the gym was not systematically assessed.

Future studies should include a controlled design (e.g. randomized controlled trials with placebo treatments), using both genders and various populations (e.g. students). Furthermore, research should analyze different exercise types, individual and group settings, exercise combined with other forms of therapy, and the long-term effects of regular exercise. Above all, researchers should discover whether participants in structured programs could sustain regular exercise independently, and whether behavioural skill training (e.g., coping planning, facilitating social support) would prolong exercise maintenance.

## Conclusions

The present pilot study delivered preliminary evidence that exercise reduces perceived stress among participants suffering from burnout, and prevents them from developing a deeper depression. This is important since burnout can be viewed as an antecedent of depressive disorders. Thus, exercise interventions might constitute a relatively simple and inexpensive alternative compared to pharmacotherapy or psychotherapy in the treatment of burnout, particularly as exercise does not only influence psychological well-being, but also impact physiological mechanisms that link burnout to cardiovascular disease and premature death.

## Competing interests

All authors declare that they have no competing interests.

## Authors’ contributions

MG contributed to the conception and design of the study, the analysis and interpretation of the data and drafted the manuscript. SB, CE, EHT and UP were involved in the interpretation of the data and contributed to the revision of the drafted manuscript. SB provided knowledge regarding statistical and methodological problems. JB conceived the study, was responsible for data collection and co-authored the methods section of this manuscript. JB also provided knowledge regarding the interpretation of the data. All authors read and approved the final manuscript.
